# 4-Chloro­anilinium (4-chloro­phen­yl)guanidinium dichloride hemihydrate

**DOI:** 10.1107/S1600536810007774

**Published:** 2010-03-10

**Authors:** Yanhua Zhang, Xiangyun Liu

**Affiliations:** a Chemistry and Chemical Engineering Department, Henan University of Urban Construction, Pingdingshan 467044, People’s Republic of China

## Abstract

In the title hydrated molecular salt, C_6_H_7_ClN^+^·C_7_H_9_ClN_3_^+^·2Cl^−^·0.5H_2_O, the water O atom lies on a crystallographic twofold axis. In the crystal, inter­molecular N—H⋯Cl and O—H⋯Cl hydrogen bonds form layers perpendicular to the *ac* plane in which both the water mol­ecule and the chloride anion are involved in connecting the layers into a three-dimensional structure.

## Related literature

For applications of guanidine-containing compounds, see: Yonehara & Otake (1966 ▶); Berlinck (1995 ▶); Gobbi & Frenking (1993 ▶). For related structures, see: Ploug-Sørenson & Andersen 1985 ▶; Kolev *et al.* (1997 ▶); Glidewell *et al.* (2005 ▶); Smith *et al.* (2005 ▶).
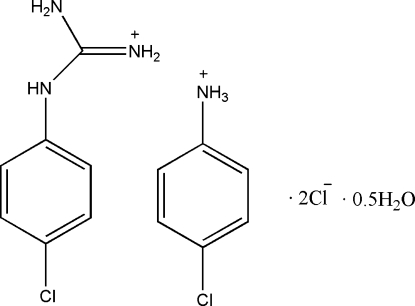


## Experimental

### 

#### Crystal data

C_6_H_7_ClN^+^·C_7_H_9_ClN_3_^+^·2Cl^−^·0.5H_2_O
*M* *_r_* = 379.11
Monoclinic, 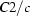

*a* = 41.297 (8) Å
*b* = 4.2089 (8) Å
*c* = 23.695 (5) Å
β = 120.164 (2)°
*V* = 3560.8 (12) Å^3^
*Z* = 8
Mo *K*α radiation
μ = 0.67 mm^−1^
*T* = 298 K
0.51 × 0.50 × 0.34 mm

#### Data collection

Bruker SMART CCD area-detector diffractometer
Absorption correction: multi-scan (*SADABS*; Sheldrick, 1996 ▶) *T*_min_ = 0.727, *T*_max_ = 0.805
8167 measured reflections
3078 independent reflections
2495 reflections with *I* > 2σ(*I*)
*R*_int_ = 0.046

#### Refinement

*R*[*F* ^2^ > 2σ(*F* ^2^)] = 0.045
*wR*(*F* ^2^) = 0.122
*S* = 1.03
3078 reflections
211 parameters
1 restraint
H atoms treated by a mixture of independent and constrained refinement
Δρ_max_ = 0.33 e Å^−3^
Δρ_min_ = −0.22 e Å^−3^

### 

Data collection: *SMART* (Bruker, 1998 ▶); cell refinement: *SAINT* (Bruker, 1999 ▶); data reduction: *SAINT*; program(s) used to solve structure: *SHELXTL* (Sheldrick, 2008 ▶); program(s) used to refine structure: *SHELXTL*; molecular graphics: *SHELXTL*; software used to prepare material for publication: *SHELXTL*.

## Supplementary Material

Crystal structure: contains datablocks global, I. DOI: 10.1107/S1600536810007774/hg2652sup1.cif

Structure factors: contains datablocks I. DOI: 10.1107/S1600536810007774/hg2652Isup2.hkl

Additional supplementary materials:  crystallographic information; 3D view; checkCIF report

## Figures and Tables

**Table 1:** Hydrogen-bond geometry (Å, °)

*D*—H⋯*A*	*D*—H	H⋯*A*	*D*⋯*A*	*D*—H⋯*A*
O1—H14*A*⋯Cl1^i^	0.82 (2)	2.36 (2)	3.1797 (17)	177 (3)
N2—H2*A*⋯Cl2^i^	0.86	2.54	3.324 (2)	152
N3—H3*A*⋯Cl2^i^	0.86	2.48	3.281 (2)	155
N4—H4*D*⋯Cl2^ii^	0.82 (6)	2.39 (5)	3.185 (3)	164 (5)
N2—H2*B*⋯Cl2^iii^	0.86	2.62	3.2457 (19)	131
N4—H4*A*⋯Cl1^iv^	0.93 (6)	2.27 (6)	3.158 (3)	160 (5)
N1—H1*A*⋯Cl1^v^	0.86	2.52	3.283 (2)	148

Symmetry codes: (i) 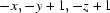; (ii) 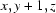; (iii) 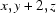; (iv) 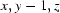; (v) 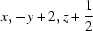.

**Fig. 1.**
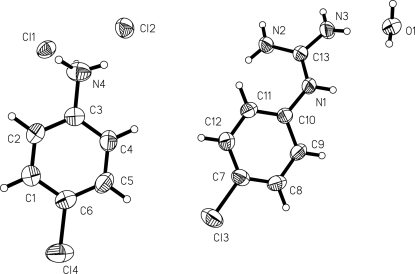
View of the title compound (I), with displacement ellipsoids drawn at the 40% probability level.

## supplementary crystallographic information

### Comment

The guanidine-containing compounds have been employed as anti-microbials and fungicides on a considerable scale(Yonehara & Otake, 1966). The drugs containing guanidine framework are not only easy to transport(Berlinck, 1995), but also make the functions of absorption and osmosis more selective due to the good solubility of their various acid salts in aqueous solution(Gobbi & Frenking, 1993). We report here the cocrystal structure of title compound.

Title compound crystallizes with one 4-chloropenylguanidinium cation , one 4-chloroanilinium cation, two chloride anion and half water molecular in the asymmetric unit (Fig. 1). All bond lengths and angles are normal (Ploug-Sørenson & Andersen, 1985; Kolev *et al.*, 1997; Glidewell *et al.*, 2005; Smith *et al.*, 2005). The forces between cations and anions consist of hydrogen bonding and ion-pairing. Intermolecular N—H···Cl and O—H···Cl hydrogen bonds form layers perpendicular to the ac plane in which both the water molecule and the chloride anion are involved in structure extension (Table 1).

### Experimental

The 4-chlorophenylguanidine (0.01 mol) was added to a solution of 4-chlorobenzenamine (0.01 mol) in ethanol (20 ml) and stirred half hour at room temperature. The mixture was adjusted to pH 2-3 with concentrated hydrochloric acid, and the desired products then precipitated, which was collected by filtration. Single crystals suitable for X-ray measurements were obtained by recrystallization from methanol and water (v/v 1:1) at room temperature for one week.

### Refinement

Hydrogen atoms bonded to O and 4-chloroanilinium N were located by difference methods and their positional and isotropic displacement parameters were refined but these were constrained in the final refinement cycles. H atoms bonded to C and 4-chlorophenylguanidinium N atoms were treated as riding atoms, with C—H distances of 0.93 Å and N—H distances of 0.86 Å and U_iso_(H) values of 1.2U_eq_(C,*N*).

### Crystal data

**Table:**

C_6_H_7_ClN^+^·C_7_H_9_ClN_3_^+^·2Cl^−^·0.5H_2_O	*F*(000) = 1560
*M**_r_* = 379.11	*D*_x_ = 1.414 Mg m^−^^3^
Monoclinic, *C*2/*c*	Mo *K*α radiation, λ = 0.71073 Å
Hall symbol: -C 2yc	Cell parameters from 2794 reflections
*a* = 41.297 (8) Å	θ = 2.6–24.3°
*b* = 4.2089 (8) Å	µ = 0.67 mm^−^^1^
*c* = 23.695 (5) Å	*T* = 298 K
β = 120.164 (2)°	Block, colorless
*V* = 3560.8 (12) Å^3^	0.51 × 0.50 × 0.34 mm
*Z* = 8	

### Data collection

**Table:**

Bruker SMART CCD area-detector diffractometer	3078 independent reflections
Radiation source: fine-focus sealed tube	2495 reflections with *I* > 2σ(*I*)
graphite	*R*_int_ = 0.046
φ and ω scans	θ_max_ = 25.0°, θ_min_ = 2.0°
Absorption correction: multi-scan (*SADABS*; Sheldrick, 1996)	*h* = −45→48
*T*_min_ = 0.727, *T*_max_ = 0.805	*k* = −5→4
8167 measured reflections	*l* = −27→28

### Refinement

**Table:**

Refinement on *F*^2^	Primary atom site location: structure-invariant direct methods
Least-squares matrix: full	Secondary atom site location: difference Fourier map
*R*[*F*^2^ > 2σ(*F*^2^)] = 0.045	Hydrogen site location: inferred from neighbouring sites
*wR*(*F*^2^) = 0.122	H atoms treated by a mixture of independent and constrained refinement
*S* = 1.03	*w* = 1/[σ^2^(*F*_o_^2^) + (0.0657*P*)^2^ + 0.9195*P*] where *P* = (*F*_o_^2^ + 2*F*_c_^2^)/3
3078 reflections	(Δ/σ)_max_ = 0.001
211 parameters	Δρ_max_ = 0.33 e Å^−^^3^
1 restraint	Δρ_min_ = −0.22 e Å^−^^3^

### Special details

**Table:**

Geometry. All esds (except the esd in the dihedral angle between two l.s. planes) are estimated using the full covariance matrix. The cell esds are taken into account individually in the estimation of esds in distances, angles and torsion angles; correlations between esds in cell parameters are only used when they are defined by crystal symmetry. An approximate (isotropic) treatment of cell esds is used for estimating esds involving l.s. planes.
Refinement. Refinement of *F*^2^ against ALL reflections. The weighted *R*-factor wR and goodness of fit *S* are based on *F*^2^, conventional *R*-factors *R* are based on *F*, with *F* set to zero for negative *F*^2^. The threshold expression of *F*^2^ > σ(*F*^2^) is used only for calculating *R*-factors(gt) etc. and is not relevant to the choice of reflections for refinement. *R*-factors based on *F*^2^ are statistically about twice as large as those based on *F*, and *R*- factors based on ALL data will be even larger.

### Fractional atomic coordinates and isotropic or equivalent isotropic displacement parameters (Å^2^)

**Table:**

	*x*	*y*	*z*	*U*_iso_*/*U*_eq_	
Cl1	0.073762 (19)	0.49131 (14)	0.32708 (3)	0.0542 (2)	
Cl2	0.062005 (17)	−0.51049 (14)	0.49527 (3)	0.0496 (2)	
Cl3	0.20900 (2)	0.7771 (2)	0.72411 (4)	0.0787 (3)	
Cl4	0.26245 (2)	0.1128 (3)	0.59514 (5)	0.0943 (3)	
O1	0.0000	0.9261 (7)	0.7500	0.0535 (6)	
H14A	−0.0188 (6)	0.815 (7)	0.7317 (14)	0.074 (10)*	
N1	0.06411 (5)	1.1656 (6)	0.69506 (9)	0.0560 (6)	
H1A	0.0648	1.1745	0.7319	0.067*	
N2	0.02658 (6)	1.2676 (6)	0.58408 (9)	0.0583 (6)	
H2A	0.0051	1.3206	0.5519	0.070*	
H2B	0.0450	1.2324	0.5777	0.070*	
N3	0.00277 (6)	1.2948 (6)	0.65192 (10)	0.0614 (6)	
H3A	−0.0186	1.3477	0.6193	0.074*	
H3B	0.0055	1.2777	0.6902	0.074*	
N4	0.09760 (8)	−0.0049 (7)	0.43996 (17)	0.0644 (7)	
C1	0.19749 (9)	−0.0652 (8)	0.48977 (15)	0.0711 (8)	
H1B	0.2122	−0.1407	0.4731	0.085*	
C2	0.15937 (8)	−0.0937 (7)	0.45442 (14)	0.0649 (7)	
H2C	0.1480	−0.1909	0.4137	0.078*	
C3	0.13796 (7)	0.0206 (5)	0.47893 (13)	0.0473 (6)	
C4	0.15458 (8)	0.1594 (7)	0.53908 (13)	0.0604 (7)	
H4C	0.1399	0.2353	0.5557	0.073*	
C5	0.19272 (8)	0.1877 (7)	0.57510 (13)	0.0649 (7)	
H5A	0.2040	0.2820	0.6161	0.078*	
C6	0.21393 (8)	0.0766 (6)	0.55030 (13)	0.0569 (7)	
C7	0.16604 (7)	0.8951 (6)	0.71382 (12)	0.0500 (6)	
C8	0.16519 (8)	1.0740 (6)	0.76169 (13)	0.0572 (7)	
H8A	0.1873	1.1340	0.7988	0.069*	
C9	0.13103 (7)	1.1630 (7)	0.75378 (12)	0.0567 (7)	
H9A	0.1301	1.2838	0.7858	0.068*	
C10	0.09795 (6)	1.0742 (6)	0.69845 (11)	0.0429 (5)	
C11	0.09949 (7)	0.8967 (6)	0.65113 (11)	0.0482 (6)	
H11A	0.0775	0.8364	0.6137	0.058*	
C12	0.13380 (7)	0.8084 (6)	0.65932 (13)	0.0522 (6)	
H12A	0.1348	0.6886	0.6273	0.063*	
C13	0.03129 (7)	1.2401 (6)	0.64323 (11)	0.0455 (6)	
H4D	0.0856 (16)	0.130 (14)	0.446 (3)	0.17 (2)*	
H4B	0.0890 (13)	−0.130 (12)	0.457 (2)	0.14 (2)*	
H4A	0.0872 (15)	−0.110 (14)	0.400 (3)	0.18 (2)*	

### Atomic displacement parameters (Å^2^)

**Table:**

	*U*^11^	*U*^22^	*U*^33^	*U*^12^	*U*^13^	*U*^23^
Cl1	0.0552 (4)	0.0610 (4)	0.0393 (4)	−0.0094 (3)	0.0185 (3)	0.0031 (3)
Cl2	0.0461 (4)	0.0655 (4)	0.0406 (3)	0.0053 (3)	0.0243 (3)	0.0064 (3)
Cl3	0.0503 (4)	0.1045 (6)	0.0858 (6)	0.0173 (4)	0.0377 (4)	0.0099 (5)
Cl4	0.0518 (5)	0.1166 (7)	0.0872 (6)	−0.0050 (5)	0.0146 (4)	−0.0111 (5)
O1	0.0472 (16)	0.0582 (16)	0.0483 (15)	0.000	0.0191 (13)	0.000
N1	0.0399 (12)	0.0949 (17)	0.0320 (10)	0.0124 (12)	0.0172 (9)	0.0028 (11)
N2	0.0430 (12)	0.0954 (17)	0.0388 (11)	0.0095 (11)	0.0223 (10)	0.0160 (11)
N3	0.0434 (12)	0.1009 (18)	0.0432 (11)	0.0155 (12)	0.0241 (10)	0.0109 (12)
N4	0.0506 (15)	0.0518 (14)	0.081 (2)	−0.0028 (12)	0.0254 (15)	0.0001 (14)
C1	0.0595 (19)	0.093 (2)	0.0647 (18)	−0.0002 (17)	0.0344 (16)	−0.0173 (17)
C2	0.0610 (18)	0.0809 (19)	0.0507 (16)	−0.0073 (16)	0.0266 (14)	−0.0191 (15)
C3	0.0495 (15)	0.0382 (12)	0.0510 (14)	−0.0007 (10)	0.0230 (12)	0.0069 (11)
C4	0.0640 (18)	0.0716 (18)	0.0497 (15)	0.0065 (15)	0.0315 (14)	−0.0041 (14)
C5	0.0687 (19)	0.0749 (19)	0.0416 (14)	0.0009 (16)	0.0207 (14)	−0.0103 (13)
C6	0.0506 (15)	0.0593 (16)	0.0502 (15)	−0.0015 (13)	0.0174 (13)	0.0017 (13)
C7	0.0422 (14)	0.0550 (14)	0.0540 (15)	0.0063 (12)	0.0251 (12)	0.0086 (13)
C8	0.0439 (15)	0.0668 (17)	0.0461 (15)	0.0004 (13)	0.0116 (12)	−0.0033 (13)
C9	0.0479 (15)	0.0759 (18)	0.0364 (13)	0.0103 (14)	0.0137 (12)	−0.0065 (13)
C10	0.0394 (13)	0.0513 (13)	0.0351 (12)	0.0064 (11)	0.0167 (11)	0.0063 (10)
C11	0.0423 (14)	0.0536 (13)	0.0414 (13)	−0.0010 (12)	0.0155 (11)	−0.0049 (11)
C12	0.0545 (16)	0.0547 (15)	0.0514 (15)	0.0049 (12)	0.0295 (13)	−0.0057 (12)
C13	0.0404 (13)	0.0579 (14)	0.0382 (12)	0.0012 (11)	0.0197 (11)	0.0034 (11)

### Geometric parameters (Å, °)

**Table:**

Cl3—C7	1.738 (2)	C1—H1B	0.9300
Cl4—C6	1.740 (3)	C2—C3	1.366 (4)
O1—H14A	0.820 (17)	C2—H2C	0.9300
N1—C13	1.331 (3)	C3—C4	1.364 (4)
N1—C10	1.412 (3)	C4—C5	1.369 (4)
N1—H1A	0.8600	C4—H4C	0.9300
N2—C13	1.320 (3)	C5—C6	1.359 (4)
N2—H2A	0.8600	C5—H5A	0.9300
N2—H2B	0.8600	C7—C12	1.359 (4)
N3—C13	1.314 (3)	C7—C8	1.377 (4)
N3—H3A	0.8600	C8—C9	1.379 (4)
N3—H3B	0.8600	C8—H8A	0.9300
N4—C3	1.448 (4)	C9—C10	1.387 (3)
N4—H4D	0.82 (6)	C9—H9A	0.9300
N4—H4B	0.84 (5)	C10—C11	1.375 (3)
N4—H4A	0.93 (6)	C11—C12	1.381 (3)
C1—C2	1.367 (4)	C11—H11A	0.9300
C1—C6	1.377 (4)	C12—H12A	0.9300
			
C13—N1—C10	129.5 (2)	C6—C5—C4	119.4 (3)
C13—N1—H1A	115.2	C6—C5—H5A	120.3
C10—N1—H1A	115.2	C4—C5—H5A	120.3
C13—N2—H2A	120.0	C5—C6—C1	120.8 (3)
C13—N2—H2B	120.0	C5—C6—Cl4	120.0 (2)
H2A—N2—H2B	120.0	C1—C6—Cl4	119.2 (2)
C13—N3—H3A	120.0	C12—C7—C8	120.8 (2)
C13—N3—H3B	120.0	C12—C7—Cl3	120.0 (2)
H3A—N3—H3B	120.0	C8—C7—Cl3	119.2 (2)
C3—N4—H4D	116 (4)	C7—C8—C9	119.0 (2)
C3—N4—H4B	112 (3)	C7—C8—H8A	120.5
H4D—N4—H4B	85 (5)	C9—C8—H8A	120.5
C3—N4—H4A	119 (3)	C8—C9—C10	120.6 (2)
H4D—N4—H4A	120 (5)	C8—C9—H9A	119.7
H4B—N4—H4A	95 (4)	C10—C9—H9A	119.7
C2—C1—C6	119.3 (3)	C11—C10—C9	119.3 (2)
C2—C1—H1B	120.3	C11—C10—N1	123.5 (2)
C6—C1—H1B	120.3	C9—C10—N1	117.2 (2)
C3—C2—C1	120.0 (3)	C10—C11—C12	119.8 (2)
C3—C2—H2C	120.0	C10—C11—H11A	120.1
C1—C2—H2C	120.0	C12—C11—H11A	120.1
C4—C3—C2	120.1 (3)	C7—C12—C11	120.5 (2)
C4—C3—N4	120.8 (3)	C7—C12—H12A	119.7
C2—C3—N4	119.1 (3)	C11—C12—H12A	119.7
C3—C4—C5	120.4 (2)	N3—C13—N2	119.1 (2)
C3—C4—H4C	119.8	N3—C13—N1	118.3 (2)
C5—C4—H4C	119.8	N2—C13—N1	122.6 (2)
			
C6—C1—C2—C3	0.6 (5)	C7—C8—C9—C10	−0.1 (4)
C1—C2—C3—C4	−0.9 (4)	C8—C9—C10—C11	−0.3 (4)
C1—C2—C3—N4	178.4 (3)	C8—C9—C10—N1	177.9 (2)
C2—C3—C4—C5	0.5 (4)	C13—N1—C10—C11	−34.1 (4)
N4—C3—C4—C5	−178.8 (3)	C13—N1—C10—C9	147.8 (3)
C3—C4—C5—C6	0.1 (4)	C9—C10—C11—C12	0.2 (4)
C4—C5—C6—C1	−0.4 (4)	N1—C10—C11—C12	−177.8 (2)
C4—C5—C6—Cl4	179.6 (2)	C8—C7—C12—C11	−0.4 (4)
C2—C1—C6—C5	0.0 (5)	Cl3—C7—C12—C11	179.0 (2)
C2—C1—C6—Cl4	−180.0 (2)	C10—C11—C12—C7	0.1 (4)
C12—C7—C8—C9	0.4 (4)	C10—N1—C13—N3	174.8 (3)
Cl3—C7—C8—C9	−179.0 (2)	C10—N1—C13—N2	−6.7 (4)

### Hydrogen-bond geometry (Å, °)

**Table:**

*D*—H···*A*	*D*—H	H···*A*	*D*···*A*	*D*—H···*A*
O1—H14A···Cl1^i^	0.82 (2)	2.36 (2)	3.1797 (17)	177 (3)
N2—H2A···Cl2^i^	0.86	2.54	3.324 (2)	152
N3—H3A···Cl2^i^	0.86	2.48	3.281 (2)	155
N4—H4D···Cl2^ii^	0.82 (6)	2.39 (5)	3.185 (3)	164 (5)
N2—H2B···Cl2^iii^	0.86	2.62	3.2457 (19)	131
N4—H4A···Cl1^iv^	0.93 (6)	2.27 (6)	3.158 (3)	160 (5)
N1—H1A···Cl1^v^	0.86	2.52	3.283 (2)	148

Symmetry codes: (i) −*x*, −*y*+1, −*z*+1; (ii) *x*, *y*+1, *z*; (iii) *x*, *y*+2, *z*; (iv) *x*, *y*−1, *z*; (v) *x*, −*y*+2, *z*+1/2.
